# QEEG Spectral and Coherence Assessment of Autistic Children in Three Different Experimental Conditions

**DOI:** 10.1007/s10803-013-1909-5

**Published:** 2013-09-19

**Authors:** Calixto Machado, Mario Estévez, Gerry Leisman, Robert Melillo, Rafael Rodríguez, Phillip DeFina, Adrián Hernández, Jesús Pérez-Nellar, Rolando Naranjo, Mauricio Chinchilla, Nicolás Garófalo, José Vargas, Carlos Beltrán

**Affiliations:** 1 Department of Clinical Neurophysiology, Institute of Neurology and Neurosurgery, 29 y D, Vedado, 10400 Havana, La Habana Cuba; 2 Institute for Brain and Rehabilitation Sciences, Nazareth, Israel; 3 F. R. Carrick Institute for Clinical Ergonomics, Rehabilitation, and Applied Neurosciences (CERAN), Garden City, NY USA; 4 Biomedical Engineering, ORT-Braude College of Engineering, Carmiel, Israel; 5 International Center for Neurological Restoration, Havana, Cuba; 6 International Brain Research Foundation, Flanders, NJ USA; 7 Service of Neurology, Hermanos Ameijeiras Hospital, Havana, Cuba

**Keywords:** Autism, Autism spectrum disorder, EEG, QEEG

## Abstract

We studied autistics by quantitative EEG spectral and coherence analysis during three experimental conditions: basal, watching a cartoon with audio (V–A), and with muted audio band (VwA). Significant reductions were found for the absolute power spectral density (PSD) in the central region for delta and theta, and in the posterior region for sigma and beta bands, lateralized to the right hemisphere. When comparing VwA versus the V–A in the midline regions, we found significant decrements of absolute PSD for delta, theta and alpha, and increments for the beta and gamma bands. In autistics, VwA versus V–A tended to show lower coherence values in the right hemisphere. An impairment of visual and auditory sensory integration in autistics might explain our results.

## Introduction

Autism spectrum disorder (ASD) is a complex neurodevelopmental disorder syndrome which is clinically characterized by lessened social interaction, language impairments, behavioral stereotypes, and varied cognitive deficits (Berg and Plioplys 2012; McPartland et al. 2011; McPartland and Volkmar 2012; Ratajczak 2011; South et al. 2012; Wing et al. 2011; Zappella 2012).

Quantitative EEG (QEEG), has been used for assessing autistic children by both spectral and coherence calculation methods, and although methodology and experimental paradigms have varied among these reports, they have shown some reliably abnormal EEG characteristics in these individuals (Bosl et al. 2011; Duffy and Als 2012; Kouijzer et al. 2012; Mathewson et al. 2012; Thatcher et al. 2009; Tierney et al. 2012).

Regarding spectral analysis, Cantor et al. (1986), Cantor and Chabot (2009) reported reduced Alpha activity, meanwhile other authors indicated decrement of this EEG frequency band, bilaterally and frontally (Ogawa et al. 1982). Chan et al. demonstrated that children with autism showed significantly higher relative Delta and lower relative Alpha activities (Chan and Leung 2006; Chan et al. 2007). Other authors have stated that alignment of the induced Gamma oscillations improves sensitivity in differentiation of EEG responses to emotional facial stimuli in autism, and that an excess of high frequency EEG, suggests an imbalance in the excitation-inhibition homeostasis in the cortex (Orekhova et al. 2007).

Although several papers have appeared on the assessment of EEG coherence, there are contradictory results among authors. Moreover, it is also necessary to address how coherence is calculated from EEG electrode sites, which will undoubtedly yield differing EEG coherence values (Duffy and Als 2012; Lazar et al. 2010; Lazarev et al. 2010; VarandaCde and Fernandes 2011).

Up-to-date brain theories suggest that the coordinated integration of transient activity patterns in diverse brain regions suggest a possible temporal binding deficit in autism, indicating the needs for a proper modeling of white matter anatomic and functional connectivity (Keehn et al. 2012; Leisman et al. 2012; Melillo and Leisman 2009; Testa-Silva et al. 2012; Vissers et al. 2012).

EEG/MEG methodology studies offer the necessary temporal resolution to completely describe the functional connectivity within and between both local and large scale coordinated networks (Machado et al. 2004; Mathewson et al. 2012). EEG coherence is mostly sensitive to changes in connectivity, and can be understood as some degree of the oscillatory synchrony (phase locking) between two brain regions (Duffy and Als 2012; Lazar et al. 2010; Mathewson et al. 2012). Therefore, several authors have defended that EEG coherence is a powerful tool for assessing functional connectivity in autism (Duffy and Als 2012; Lazar et al. 2010; Lazarev et al. 2010; VarandaCde and Fernandes 2011).

An important handicap to assess autistic subjects by QEEG methodology (both spectral and coherence analysis) is to record EEG either in the eyes-closed or eyes-open resting condition, as children diagnosed with autism do not usually cooperate during the experimental session. It is well known from literature that the recording of EEG in eyes-closed or eyes-open resting conditions endows cortical processing of visual input, producing differences in activation between eyes-closed and eyes-open resting conditions in all EEG bands, rather than just the simple increase in arousal level shown in alpha band (Barry et al. 2009).

Moreover, to develop our experimental design we took in consideration that atypical sensory-based behaviors are an ever-present feature of sensory information processing in ASD (Anderson et al. 2010; Marco et al. 2011; Ouimet et al. 2012; Tecchio et al. 2003). Regarding auditory stimuli, several authors advocate that children with autism’s capability to process auditory sensory stimuli is predominantly impaired, and there exists a dysfunction of cortical auditory discrimination (Anderson et al. 2010; Foss-Feig et al. 2010; Kwakye et al. 2011; Marco et al. 2011; O’Connor 2012; Ouimet et al. 2012; Stagnitti et al. 2012).

Thus, the aim of the present study was to assess autistic children, compared with a control group, by QEEG spectral and coherence analysis, while recording EEG during an eyes-open period, in three experimental conditions: basal resting condition, watching a popular cartoon with audio, and watching the cartoon with a muted audio band.

## Methods

### Participants

Two groups of right-handed participants, 11 children diagnosed with autism, 7 (63.6 %) males and 4 (36.4 %) females, and 14 healthy control subjects, 9 (64.3 %) males and 5 (35.7 %) females, without statistically significant differences in age (ASD group: 70.3 ± 29.32 months; control group: 66.68 ± 29.6), were included in this study.

Children with autism were selected from the outpatient child neurology clinic of the Institute of Neurology and Neurosurgery, Havana, Cuba, who were blindly clinically examined by two child neurologists, and diagnosed as complaining an autistic disorder, based on DSM-IV criteria (American Psychiatric Association 2000). All of them had a classical autistic triad of impairments in social interaction, communication and imagination (Belmonte et al. 2009; Gadow and Drabick 2012; Rapin and Tuchman 2008; Silver and Rapin 2012; Zappella 2012), with relatively intact verbal functions and with IQs over 85 (Charman et al. 2011). They did not have epileptic symptoms and neurologic abnormalities other than those directly related to autism. The ASD subjects were free of drug treatment.

Inclusion criteria for the Control group were based on: a history of uneventful prenatal, perinatal, and neonatal periods; no disorders of consciousness, no history of central or peripheral nervous system disease, of head injury with cerebral symptoms, convulsive episodes, paroxysmal, headache, enuresis or encopresis after the fourth birthday, tics, stuttering, pavor-nocturnus, or any psychiatric, behavior or drug related disorder. All children showed normal academic achievement (American Psychiatric Association 2000; Rapin and Tuchman 2008). Individuals were excluded from the control group if any spike wave activity was present in the EEG.

The Ethics Committee of the Institute of Neurology and Neurosurgery approved this research and the participants’ relatives or persons responsible gave informed consent to participate in the study.

Participants were studied in a laboratory with controlled temperature from 24 to 26 °C, with noise attenuation and dimmed lights. One parent and a trained technician were present during the recording sessions, as well as the clinician in charge. All participants seated in a comfortable chair, and prior to the experimental session, they were familiarized with the room and experimental set to achieve better collaboration. The standard test consisted of three parts. During the first one, participants looked to the center of a shut TV monitor with a colored green dot in the center of the screen. Participants were instructed to fixate the dot trying not to change their gaze or move their heads or any other part of the body. This part of the experimental session was referred to as *control* (C) and had duration of at least 10 min. Later, a popular cartoon for children in our country was presented for approximately 5 min, and the participants were asked to pay attention to it, and avoid unnecessary movements, particularly of the head. The original cartoon’s audio was turned to a moderate intensity level. This experimental section was referred as *“video and audio”* (*V*–*A*). During the third and last section, which lasted also approximately 5 min, the cartoon audio band was muted and the subjects were asked to continue paying attention to the movie. The audio was always muted at a specific selected moment of the cartoon story, assuring that the presented audio-on audio-off segments were the same for all subjects. The audio track included conversation in Spanish among cartoon characters, which was important for understanding the story. This section was referred as *“video without audio” (VwA).* All sessions were video monitored to evaluate facial expressions and other signs of emotional reactions.

### EEG Recordings

EEG was recorded from 19 standard locations over the scalp according to the 10–20 system: Fp1, Fp2, F3, F4, F7, F8, T3, T4, C3, C4, P3, P4, T5, T6, O1, O2, Fz, Cz, and Pz. Gold-cup scalp electrodes applied with collodion were fixed, after a careful cleaning of the skin, using a conductor paste, and connected to the input box of the digital EEG system (Medicid-05, Neuronic, S.A.). Monopolar leads were employed, using linked ears as a reference. EEG technical parameters were: gain 20,000, pass-band filters 0.1–70 Hz, “notch” filter at 60 Hz, noise level of 2 μV (root mean square), sampling frequency 200 Hz, and electrode–skin impedance never higher than 5 KΩ. Electrodes were placed over the superior and inferior rim to record eye movement artifacts for easing to detect them in the EEG records. Two experts visually inspected the records to select free of artifacts EEG segments with a total duration of no less than 65 s for each experimental section, which were later exported to an ASCII file, and stored for further quantitative analysis.

### EEG Pre-processing

The EEG values of every one of the 19 leads were submitted off-line to a previous pre-processing set of actions consisting of: (a) subtraction of the mean value of the sequence of EEG values to diminish the effect of the DC component of the time series; (b) application of a non-linear median filter (three-points window) to exclude outliers or abnormally relatively high amplitude values; (Lin et al. 2010) (c) standard linear detrending to avoid any possible drifts in the series; (d) highpass digital filtering (low cutoff frequency of 0.5 Hz); (e) lowpass digital filtering (high cutoff frequency of 55 Hz) using a six order Butterworth filter. For both filtering processes it was applied an algorithm developed by The MathWorks Inc., which after filtering the data in the forward direction, reverses the filtered sequence and runs it back through the filter producing a zero-phase distortion effect, included in the function “filtfilt.m” of Matlab (Aoude et al. 2006).

### QEEG Spectral Analysis

EEG samples contained in the ASCII files previously described, were imported by a specifically tailored software tool developed with Matlab version 7.10.0.499 R2010a (The Mathworks, Inc.). This program included different actions including: estimation of the power spectral densities (PSD) for every EEG lead, computation of different spectral indices, and coherence calculation, and finally an output of these results to a database developed with Microsoft Access.

#### Grouping of EEG Leads for Spectral Analysis

An anterior left region was considered, including the EEG leads Fp1, F3, and F7. A corresponding anterior right region consisted of the Fp2, F4, and F8 derivations. A central left region was comprised by the C3, and the T3 leads, while a central right region included the C4, and the T4 derivations. A posterior left region included the P3, O1, and T5 leads, and a posterior right region was integrated with the P4, O2, and T6 derivations. Finally, a midline region was defined including the Fz, Cz, and Pz leads.

#### Computation of PSD and the Spectral Indices

The first 12,288 samples of the EEG values of each EEG lead were submitted to a spectral analysis implemented with the Welch periodogram method, using a Hann window to avoid as possible the leakage effect. Windows of 1,024 samples (5.12 s), overlapped every 512 samples, were used by this algorithm yielding 23 consecutive windows, and the estimated PSD results for every discrete spectral frequency were averaged to obtain the global smoothed spectrum for each EEG lead. The spectral resolution for this process was 1/5.12 s, or approximately 0.1953125 Hz. The first 6 discrete frequencies, including the DC or zero frequency were discarded, and the discrete frequencies were submitted to integration within the limits selected for the different EEG bands, From these discrete frequencies were included 12 for the Delta EEG band (1.17–3.5 Hz), 22 for Theta (3.5–7.5 Hz), 19 for Alpha (7.5–11 Hz), 21 for Sigma (11–15 Hz), 53 for Beta (15–25 Hz) and 154 for Gamma (25–55 Hz). The PSD for the EEG in each band were also measured in normalized units using the standard procedure, calculating the percent of the PSD with respect to the total PSD of the whole spectral range (Lazar et al. 2010; Leveille et al. 2010).

### EEG Coherence Calculation

The function of quadratic coherence (Coben et al. 2008) was calculated as the cross-spectrum, normalized by the power spectra of the two leads to be considered using the expression:

$$ Coh_{L1L2} (\omega ) = \frac{{\left| {C_{L1L2} (\omega )} \right|^{2} }}{{P_{L1L1} (\omega )\,\,P_{L2L2} (\omega )}} $$

where “C” is the cross-spectrum of the EEG time series; “L1” is one of the two EEG leads; “L2” is the other lead; “PL1L1” and “PL2L2” are the power spectra of both EEG time series. The Matlab function “mscohere.m”, was included in our Matlab program. EEG segments were defined including 1,024 samples with an overlap (50 %) of 512 samples. Finally, 12 segments were included (12,288 samples, 61.44 s). To each one a Hann window was applied for calculations of the power spectral estimations of individual EEG leads, using the Welch periodogram. The method to calculate the spectral resolution and the different EEG bands was the same, previously described for EEG spectral analysis.

Coherence values for the discrete frequencies corresponding to every EEG band were averaged for further statistical analysis previous a Zeta Fisher transformation using the expression (Hoel 1960):

$$ Zeta\_Coh\_Df = 0.5*\log_{n} \frac{(1 + ValueCoh)}{(1 - ValueCoh)}; $$

where ValueCoh is the observed coherence value and log_n_ is the natural logarithm function.

#### Grouping of EEG Leads for Coherence Analysis

**Fig. 1:**
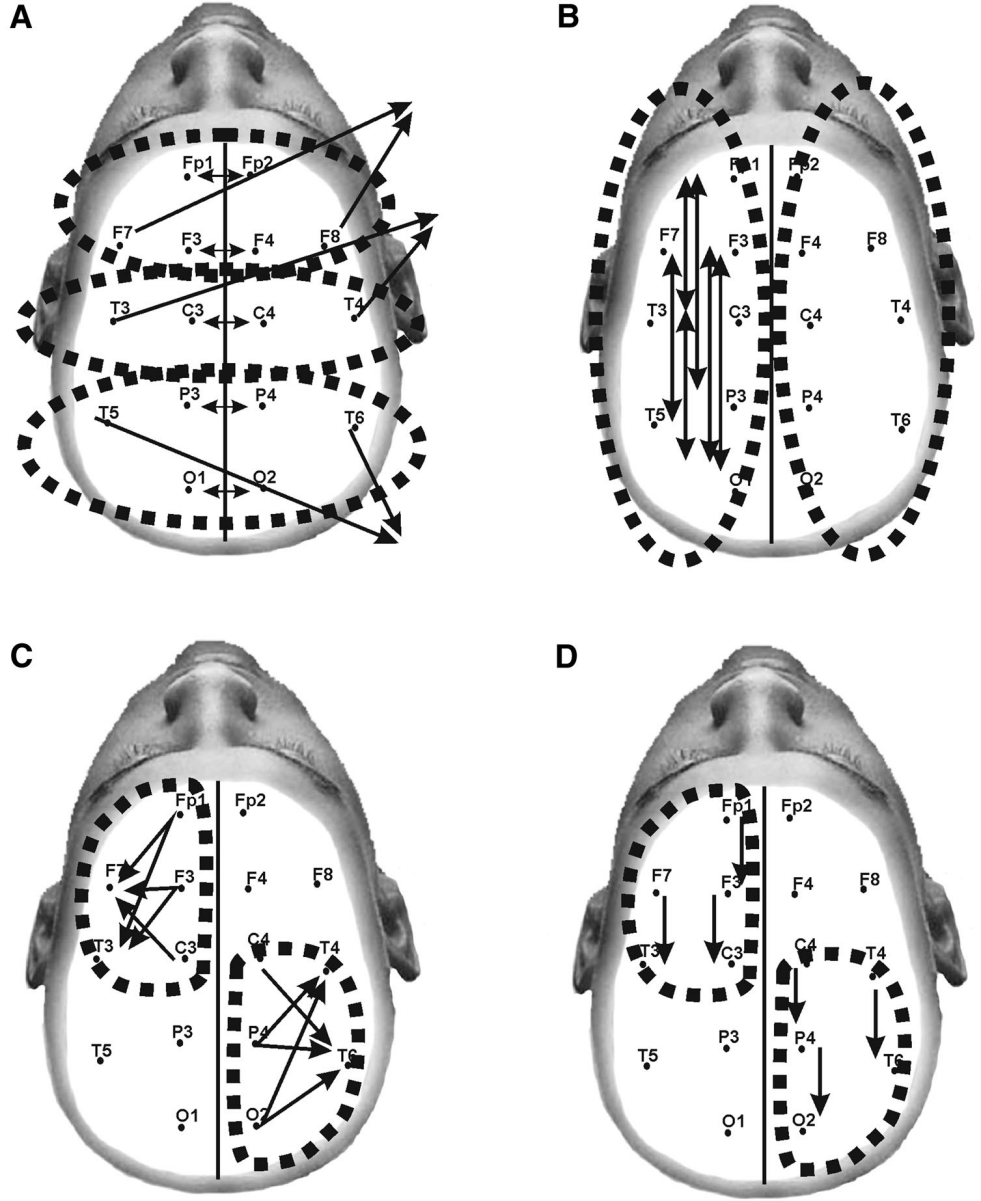
Grouping of EEG leads for coherence analysis. Four EEG lead-groupings were considered: inter-hemispheric coherence (**a**), intra-hemispheric long-range (**b**), transverse intra-hemispheric (**c**), and intra-hemispheric short-range (**d**) (Coben et al. 2008)

Four EEG lead-groupings were considered: Inter-hemispheric Coherence (Fig. 1a), Intra-hemispheric Long-Range (Fig. 1b), Transverse Intra-hemispheric (Fig. 1c), and Intra-hemispheric Short-Range (Fig. 1d) (Coben et al. 2008).

For the inter-hemispheric values of coherence a sagittal factor was considered, including posterior, central and frontal brain regions. For the anterior region the EEG Fp1–Fp2, F3–F4, and F7–F8 leads were analyzed. The EEG leads C3–C4 and T3–T4 values of coherence were considered for the central. A posterior region was also considered including the coherence values for the EEG derivations P3–P4, T5–T6, and O1–O2.

For the transverse intra-hemispheric coherence, four regions were considered: an anterior right for the EEG leads Fp2–F8, Fp2–T4, F4–F8, F4–T4, and C4–F8; an anterior left region with coherence values for Fp1–F7, Fp1–T3, F3–T3, and C3–F7 leads; a posterior right region including coherence values for C4–T6, P4–T4, P4–T6, O2–T4, and O2–T6 leads; and finally a posterior left region including coherence values for C3–T5, P3–T3, P3–T5, O1–T3, and O1–T5 derivations.

For the short-range intra-hemispheric coherences four regions were also considered: an anterior right region including Fp2–F4, F4–C4, and F8–T4 leads; an anterior left including values for Fp1–F3, F3–C3, and F7–T3 derivations; a posterior right region including T3–T5, C3–P3, and P3–O1 leads; and finally a posterior right region with coherence values for T4–T6, C4–P4, and P4–O2 derivations.

### Statistical Analysis

Normal distribution for absolute PSD values were achieved through log-natural transformations and confirmed with the Shapiro–Wilk’s W test. Normalized PSD values did not need to be transformed to attain normality distributions.

Based on the general linear model assumption, an analysis of variance (ANOVA) was carried out, considering a *between factor* (*Groups: Controls and the ASD group),* and 3 *within or repeated measures factors*: *Hemisphere: Left and Right; Sagittal areas (S Areas): including anterior regions, the two central, and the two posterior regions*; and *Experimental conditions (*“ExpCond”): *control, V*–*A, and VwA*.

We focused our attention on the results of the main effects for factor Group and on thepossible interactions between the *within* factors and the factor Group. When significant interactions (*p* < 0.05) were observed we carried out the multivariate tests of Wilks’ Lambda, Pillai–Bartlett Trace, Hotelling–Lawley Trace, and Roy’s Largest Root, and only when two from these four tests confirmed the statistical significances, a post hoc test of Newmann–Keuls was finally performed to determine the possible significant differences. Following this sequential method it was possible to assess the effects of compound symmetry and sphericity. All the statistical analysis and graphical information were performed using STATISTICA version 8.0 (StatSoft Inc.).

For the statistical analysis of each set of coherence groupings a similar approach was carried out, using ANOVA with the same test design. The statistical power was assessed for testing every analysis of the several indices (spectral or coherences) and only values over 0.90 for the between factor “Groups” were accepted.

## Results

### Behavioral Expressions of Subjects During the Experimental Sessions

When the cartoon audio band was muted, our examiners did not detect any facial or other emotion behavioral sign in the autistic children, contrary to control subjects, who frequently were upset when audio was off. This was also off-line verified in the video records of the experimental sessions.

As the experimental conditions were carried out in an eyes-open condition, the children blinked spontaneously. The number of blinks was higher in the control group in all the experimental conditions, although values didn’t achieve significant values. Control subjects showed 10.3 ± 4.2 and children with autism 8.1 ± 3.7 blinks in the control experimental condition; in the V–A experimental condition controls blinked 9.6 ± 3.8 and children with autism 6.91 ± 4.2 times; in the VwA experimental condition 11.1 ± 4.3 blinks were counted for normal subjects and 7.1 ± 3.8 for the ASD group subjects.

### Spectral Analysis

#### EEG During the Resting, Calm, Relaxed Eyes-Opened Control Session

*Values of the Total PSD* No significant differences were found for the PSD values for the whole spectral EEG range: F(2, 73) = 1.014 *p* = 0.3191.

*Values of the Absolute PSD of the Different EEG Bands* No significant differences were found for the between factor *Group* corresponding to the absolute PSD values in the Delta [F(2, 73) = 2.583, *p* = 0.1146], the Theta [F(2, 73) = 1.157, *p* = 0.6823], and Sigma [F(2, 73) = 1.855, *p* = 0.1795] EEG bands. A significant reduction for the Alpha band [F(2, 73) = 5.9217, *p* = 0.019], and significant increments for the Beta [F(2, 73) = 7.150, *p* = 0.010], and Gamma [F(2, 73) = 10.652, *p* = 0.0020] bands, were found in the group of children with autism.

No significant interactions for any EEG bands were detected for the *within* factor *Hemisphere* and the *between* factor *Group*. Considering the interactions of the *within* factor *SAreas* and the *Group between* factor significant differences were only found for the Alpha, Beta and Gamma bands. A significant interaction between the *within* factor *SAreas* and the *Groupbetween* factor was only found for the Alpha band [F(4, 146) = 2.960, *p* = 0.0046] in the control group, consisting in an increment for the anterior and the central *SAreas*, and between the central and the posterior *SAreas*. A significant interaction of the within factors *SAreas* and *Hemisphere* was also found with the *between* factor Group.

*Values of the Normalized (nu) PSD of the Different EEG Bands* No significant differences were found for the between factor *Group* regarding the EEG nu_Sigma Band [F(2, 73) = 0.144, *p* = 0.706], but significant differences were detected for the rest of the bands: significant increments for the Delta [F(2, 73) = 11.501, *p* = 0.0014], Beta [F(2, 73) = 7.570, *p* = 0.009], and Gamma bands [F(2, 73 = 8.045, *p* = 0.0066], and significant decrements in the Theta [F(2, 73) = 20.844, *p* = 0.00003], and Alpha bands [F(2, 73) = 24.529, *p* = 0.00003] in the group of children with autism. No significant differences were found for the interactions between factor *Hemisphere* and factor *Group* for any EEG band, as it was also previously observed for the PSD absolute values. Nonetheless, significant differences were found between factor *SAreas* and factor *Group* for the Delta [F(4, 146) = 5.387, *p* = 0.006], and the Alpha [F(4, 146) = 11.697 *p* = 0.00003] bands.

#### EEG During the V–A Experimental Section

*Values of the Total PSD* No significant differences were found for the Total PSD (P_Tot) values for the whole spectral EEG range [F(2, 73) = 3.847, *p* = 0.05600].

*Values of the Absolute PSD of the Different EEG Bands* The Group factor did not show significant differences for the absolute PSD values for the P_Theta [F(2, 73) = 0.065, *p* = 0.8001], P_Sigma [F(2, 73) = 0.038, *p* = 0.8467], P_Beta [F(2, 73) = 0.266, *p* = 0.6084], and P_Gamma [F(2, 73) = 1.897, *p* = 0.1748] bands. Nonetheless, significant increments were found for the theP_Delta band [F(2, 73) = 6.281, *p* = 0.000…], and significant decrements for the P_Alpha [F(2, 73) = 6.649, *p* = 0.0130] were found in the ASD group. Significant interactions for the *Group* versus *SAreas* versus *Hemisphere* were also found.

*Values of the Normalized (nu) PSD of the Different EEG Bands* The G*roup* factor did not show significant differences for the nu_Beta [F(1, 48) = 0.45518, *p* = 0.505], and the nu_Gamma [F(2, 73) = 0.0028, *p* = 0.958] bands. Significant increments were found for the nu_Delta [F(2, 73) = 9.5332, *p* = 0.0033], and significant decrements for the nu_Theta [F(2, 73) = 4.747, *p* = 0.034], the nu_Alpha [F(2, 73) = 29.357, *p* = 0.000…], and for the nu_Sigma [F(2, 73) = 5.1584, *p* = 0.0276] EEG bands were encountered in the group of children with autism. Significant interactions only were found for the within factor SAreas with the factor *Group* in the nu_Alpha band.

#### EEG During the VwA Experimental Condition

*Values of the Total PSD* P_Tot values showed higher significant values in the group of children with autism considering the *Group* factor [F(2, 73) = 3.847, *p* = 0.05600]. Significant Interactions between G*roup* and *Hemisphere and SAreas factors* were not found.

*Values of the Absolute PSD of the Different EEG Bands* The *Group* factor did not show significant differences for the absolute PSD of Theta [F(2, 73) = 0.065, *p* = 0.8000], Sigma [F(2, 73) = 0.038, *p* = 0.8466], Beta [F(2, 73) = 0.266, *p* = 0.6084], and Gamma [F(2, 73) = 1.897, *p* = 0.1748] bands. Nonetheless, for the Alpha band significant reduced values were found in the group of children with autism [F(2, 73) = 6.649, *p* = 0.013].

For the Alpha band significant interactions were detected between *SAreas* versus *Hemisphere* versus *Group* factors*, and f*or the Sigma band between *Hemisphere* versus *Group* factors.

*Values of the Normalized (nu) PSD of the Different EEG Bands* For the group of children with autism significant incremented values were found for the nu_Delta band [F(2, 73) = 9.532, *p* = 0.0033], and significant reduced values for the nu_Theta [F(2, 73) = 4.747, *p* = 0.034], the nu_Alpha [F(2, 73] = 29.347, *p* = 0.000…], and the nu_Sigma [F(2, 73) = 5.158, *p* = 0.028] bands. No significant differences were found for the nu_Beta and the nu_Gamma bands, considering *Group* factor. Only for the nu_Alpha significant values were found for the interaction SAreas versus Group. Other significant interactions were not found.

#### EEG Dynamics Between Experimental Conditions

**Fig. 2:**
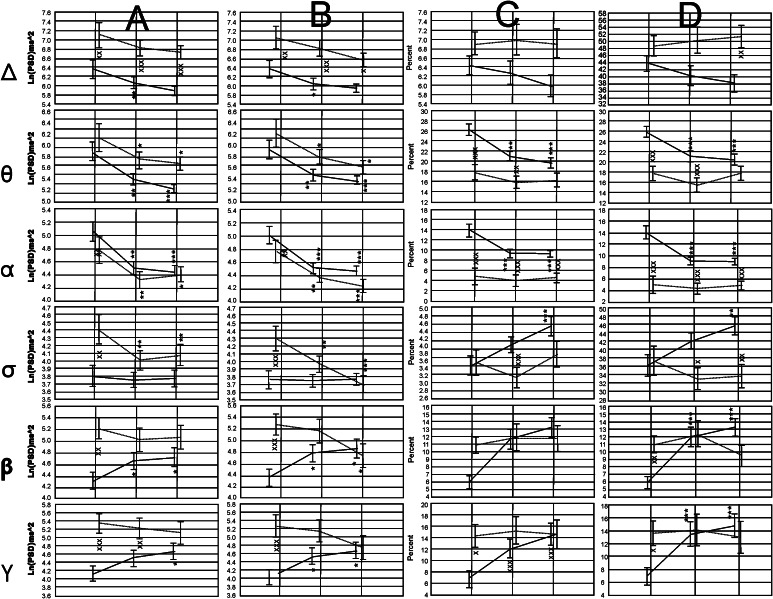
**a** Absolute values for the left anterior region. **b** Absolute values for the right anterior regions. **c** Normalized values for left anterior regions. **d** Normalized values for the right anterior regions. *Symbols* x, xx, xxx (*p* < 0.05; *p* < 0.01; *p* < 0.001) for differences between the values observed in both groups; *, **, *** (*p* < 0.05; *p* < 0.01; *p* < 0.001) for differences between the experimental conditions V–A and VwA versus the control condition; •, ••, ••• (*p* < 0.05; *p* < 0.01; *p* < 0.001) for comparisons between V–A versus VwA experimental conditions

*Anterior Regions* In the Fig. 2 a graphical collage of the results obtained for the dynamics of the absolute and normalized values of the EEG bands explored in both groups of participants is shown. In the Control group it was observed a tendency to present lower values during the experimental conditions V–A and VwA for the Delta, Theta and Alpha bands, considering absolute and normalized spectral values.

For the Sigma band, absolute values did not change during the experimental conditions, while the normalized values showed a tendency to increase. In the fast bands Beta and Gamma, a clear tendency to higher values was detected during the two experimental conditions for the absolutes and the normalized PSD values. No significant differences were found in the control group between the V–A and the VwA conditions in any spectral band.

In the ASD group significant changes were also related to the experimental conditions, but in general, there was a tendency to mimic the pattern of changes observed in the controls. For the absolute PSD the children with autism showed higher values than the Controls. For the normalized values some other patterns were detected. As for the controls group, no significant differences were found for the ASD group comparing V–A and VwA experimental conditions.

**Fig. 3:**
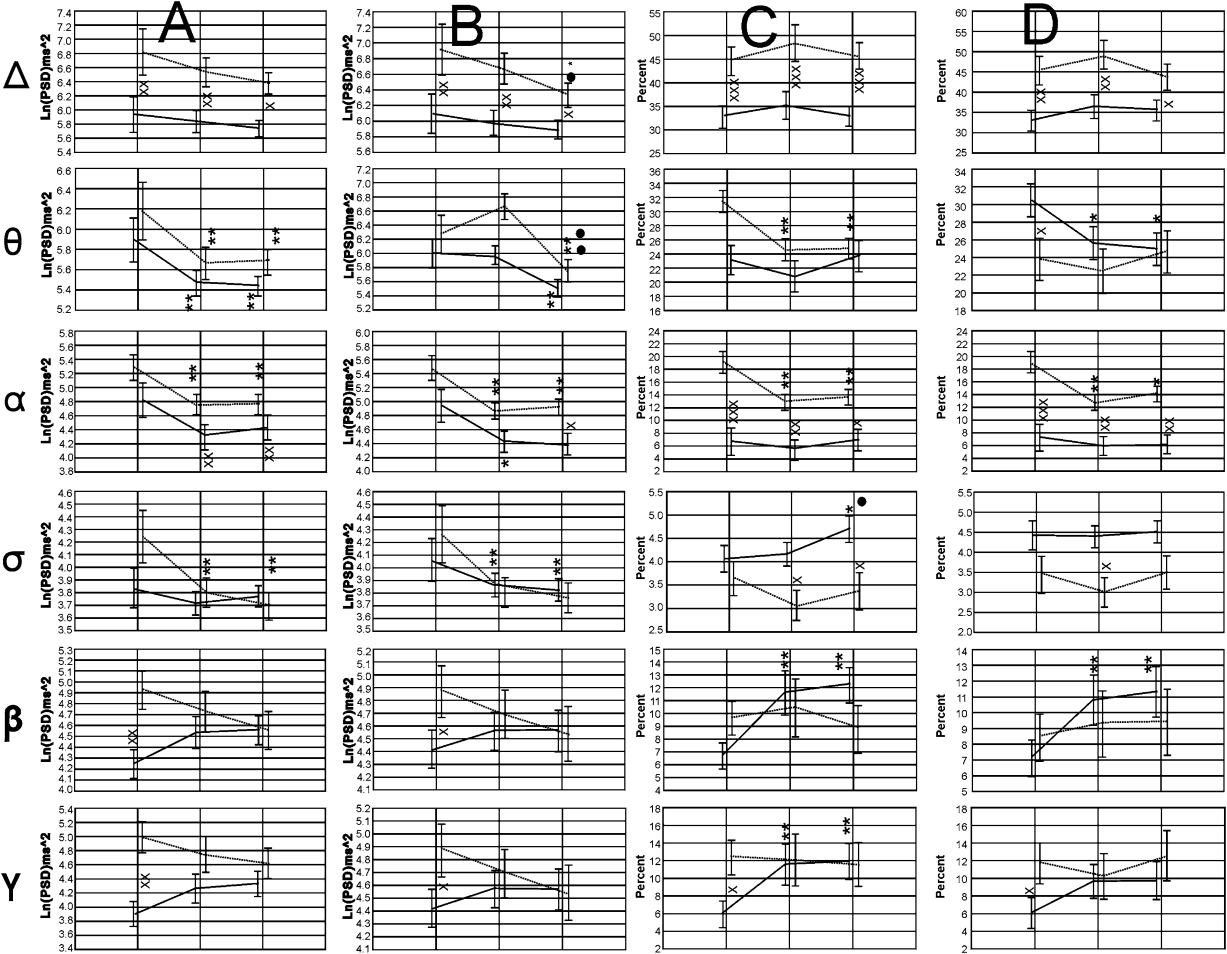
**a** Absolute values for the left central region. **b** Absolute values for the right central regions. **c** Normalized values for left anterior regions. **d** Normalized values for the right anterior regions. *Symbols* x, xx, xxx (*p* < 0.05; *p* < 0.01; *p* < 0.001) for differences between the values observed in both groups; *, **, *** (*p* < 0.05; *p* < 0.01; *p* < 0.001) for differences between the experimental conditions V–A and VwA versus the Control condition; •, ••, ••• (*p* < 0.05; *p* < 0.01; *p* < 0.001) for comparisons between V–A versus VwA experimental conditions

*Central Regions* In Fig. 3 a graphical collage of the dynamics of the EEG absolute and normalized values for the control and autistic groups, in the central region, and for the three experimental conditions, is shown. The ASD group only showed statistically significant higher absolute and normalized values for the Beta and Gamma bands in the control condition.

Absolute spectral PSD values for the Theta, alpha and Sigma band were significantly lower in the ASD group for the experimental condition V–A and VwA in comparison with control experimental values. In the control group reduced values during the V–A and VwA versus control experimental conditions were also observed for the Theta and Alpha bands.

Significant reduced values were found for the V–A state compared with the VwA condition for the absolute values of the Delta and Theta bands on the right hemisphere in the group of children with autism, and augmented normalized values of the Sigma band in the left hemisphere in the control group.

**Fig. 4:**
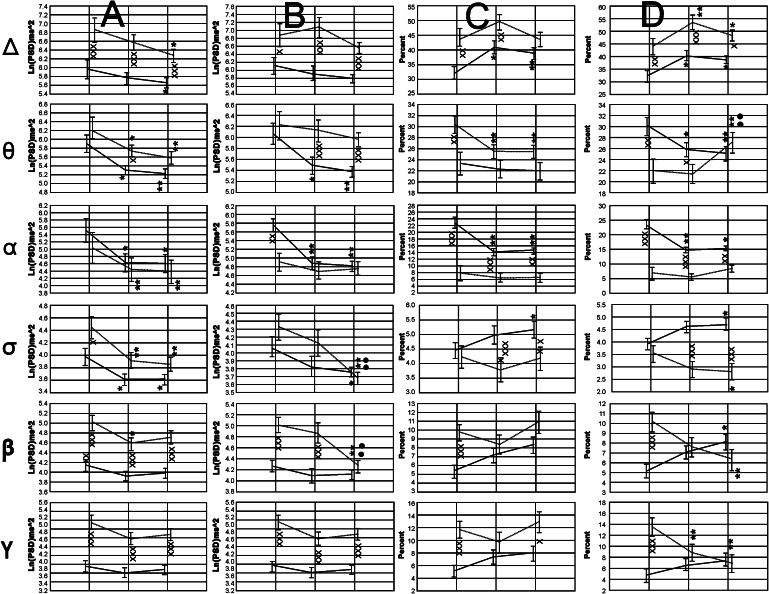
**a** Absolute values for the left posterior region. **b** Absolute values for the right posterior regions. **c** Normalized values for left posterior regions. **d** Normalized values for the right posterior regions. *Symbols* x, xx, xxx (*p* < 0.05; *p* < 0.01; *p* < 0.001) for differences between the values observed in both groups; *, **, *** (*p* < 0.05; *p* < 0.01; *p* < 0.001) for differences between the experimental conditions V–A and VwA versus the Control condition; •, ••, ••• (*p* < 0.05; *p* < 0.01; *p* < 0.001) for comparisons between V–A versus VwA experimental conditions

*Posterior Regions* In Fig. 4 a graphical collage of the dynamics of the EEG absolute and normalized values for the control and autistic groups, in the posterior region, and for the three experimental conditions, is shown. Absolute PSD values were significantly higher practically for all conditions in both hemispheres for the Delta, Theta, beta and Gamma bands in the ASD group. A similar tendency difference between both groups was observed for the normalized spectral values, but predominantly for the control condition. For the alpha and Sigma band, values expressed as normalized PSD units were significantly lower in the ASD group.

In the ASD group the V–A condition showed significant reduced values compared to the VwA state, for the absolute PSD in the Sigma and Beta bands, and incremented significant values for the Theta normalized PSD, in the right hemisphere.

In general, the dynamics of the PSD changes were less pronounced in the control group for the three experimental conditions compared with children with autism.

**Fig. 5:**
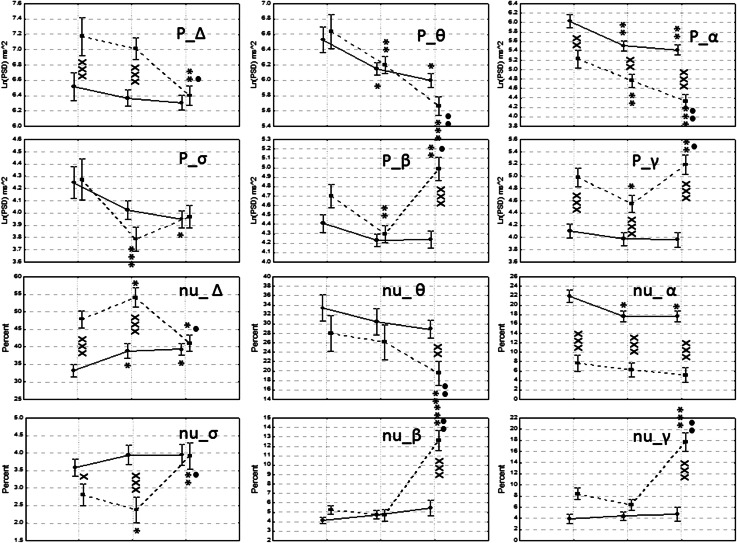
Midline regions. The *first* and *second lines* of graphics present the absolute values for bands Delta, Theta, Alpha, Sigma, Beta, and Gamma bands. The *third* and *fourth lines* of graphics show the normalized values for the corresponding band. *Symbols* x, xx, xxx (*p* < 0.05; *p* < 0.01; *p* < 0.001) for differences between the values observed in both groups; *, **, *** (*p* < 0.05; *p* < 0.01; *p* < 0.001) for differences between the experimental conditions V–A and VwA versus the Control condition; •, ••, ••• (*p* < 0.05; *p* < 0.01; *p* < 0.001) for comparisons between V–A versus VwA experimental conditions

*Midline Regions* In Fig. 5 a graphical collage of the results obtained for the dynamics of the absolute and normalized values of the EEG bands explored in both groups of participants for the midline region, in the three experimental conditions, is shown. In the Delta band, for the absolute power, the control group did not show any difference in comparisons among experimental conditions. Nonetheless, in the group of autistic children reduced significant values were found for the VwA conditions versus control values and for the V–A versus VwA condition. For the normalized spectral values, in the control group increased significant values were found for the VwA and the V–A conditions versus control values, but no differences were shown between VwA and the V–A conditions. In the group of autistic children, significant augmented values were found between the V–A condition versus control condition, and reduced values for the VwA versus control condition. Again, reduced values were found for the V–A versus VwA conditions.

In the Theta band, absolute PSD, the control group showed significant reduced values for the VwA and the V–A versus control conditions, but no differences were shown between VwA and the V–A conditions. In the group of autistic children significant reduced values were found for the V–A and the VwA versus control conditions, but the VwA also showed significant diminished values compared to the V–A conditions. For the normalized spectral values, in the control group no differences were found among experimental conditions, but the group of autistic children also showed significant augmented values for V–A versus VwA conditions. In the Alpha band, absolute PSD values, both groups of participants showed significant reduced values for the V–A and the VwA versus conditions, but only in the group of autistic children diminished values were found comparing the VwA versus V–A conditions. For the normalized spectral values only the ASD group showed reduced values for the V–A and the VwA versus control experimental conditions.

In the Sigma band, for the absolute and normalized PSD values, the control group did not show differences when comparing experimental conditions. The group of autistic children showed incremented values for the absolute PSD in the VwA versus control conditions, and for the normalized PSD values in the V–A and the VwA versus control conditions. Nonetheless, the autistic children also showed significant augmented values for V–A versus VwA conditions.

In the Beta and Gamma bands, for the absolute and normalized PSD values, the control group did not show difference in comparisons among experimental conditions. The group of autistic children showed incremented values for the absolute PSD for the V–A and the VwA versus control conditions, and for the normalized PSD values for the VwA versus control conditions. Moreover, for both the absolute and normalized PSD values the autistic children showed significant augmented values for V–A versus VwA conditions.

### EEG Coherence Results

#### Intrahemispheric Short-Range Coherence

Coherences were only found to be significant for the Between group in the Gamma band F(2, 72) = 4.9175, *p* < 0.02970 showing higher values in the autistic group.

**Fig. 6:**
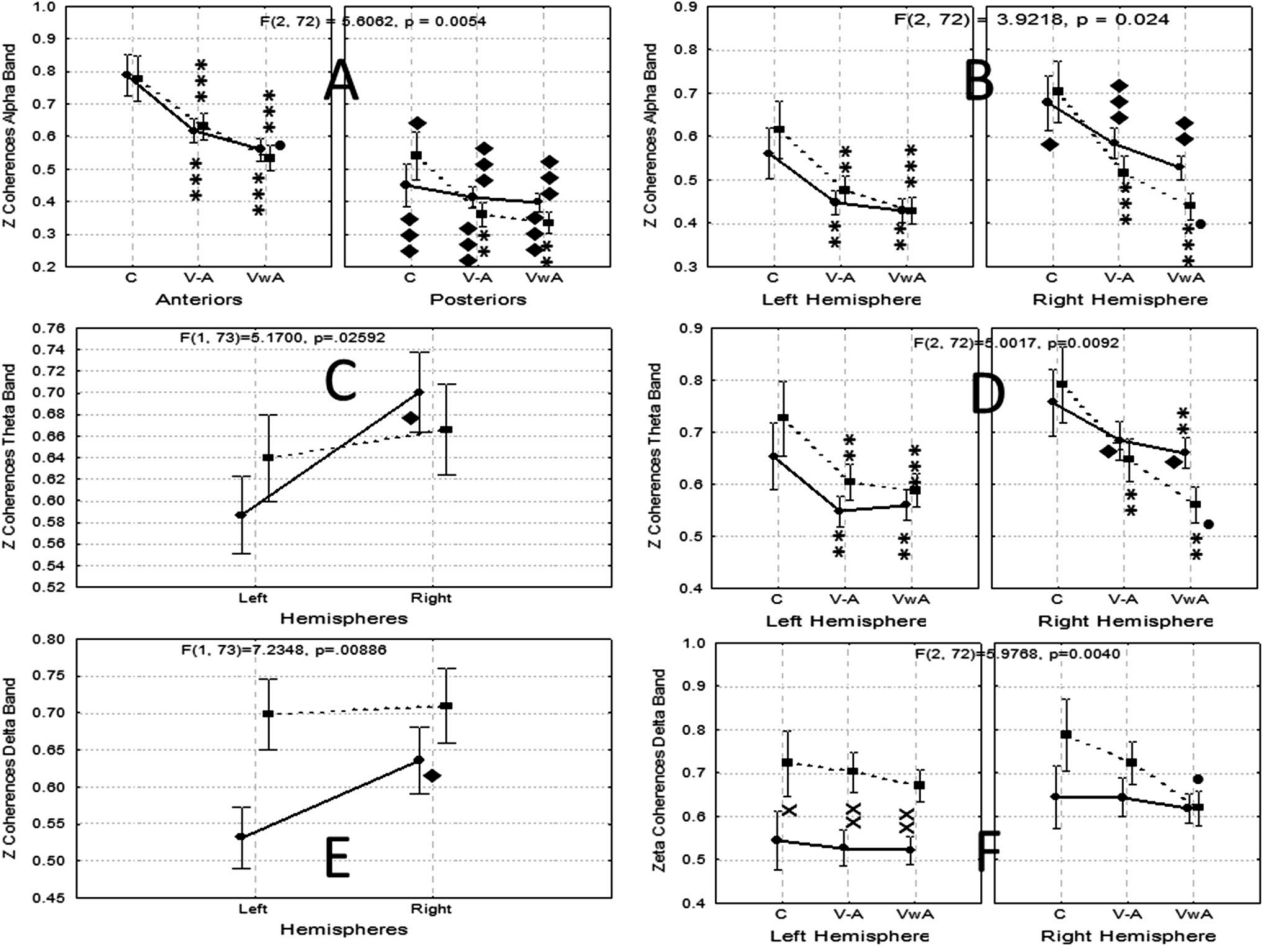
Significant changes observed in short-range intrahemispheric coherence in both groups. **a** Coherence in Alpha band, between SAreas; **b** Coherence in Alpha band between brain hemispheres; **c** Coherence in Theta band between hemispheres; **d** Coherence in Theta band between hemispheres and experimental conditions; **e** Coherence in Delta band between hemispheres; **f** Coherence in Delta band between hemispheres and experimental conditions. See more detailed information in the text; x, xx *p* < 0.05, *p* < 0.01 for comparisons between groups; **, *** *p* < 0.01, *p* < 0.001 for comparisons between experimental conditions versus control; • *p* < 0.05, *p* < 0.01 for comparisons between V–A versus VwA; ♦, ♦♦, ♦♦♦ *p* < 0.05, *p* < 0.01, *p* < 0.001 for comparisons between homologous measures between hemispheres or SAreas

Nevertheless, significant interactions were detected for the Alpha band for factors ExpCond versus SAreas versus Group [F(2, 72) = 5.6062, *p* = 0.0054] (Fig. 6a), and for factors ExpCond versus Hemis versus Group [F(2, 72) = 3.9218, *p* = 0.024] (Fig. 6b) considering the multivariate tests. In both cases the coherence values were reduced significantly during the experimental conditions V–A and VwA in comparison to those in the control condition, and even the values for the VwA condition were significantly lower in the ASD group, in comparison with the V–A condition. Significantly higher interhemispheric differences were observed in both groups in the right hemisphere. Significantly lower values were found in the posterior SAreas for both groups (Fig. 6a, b). Coherence in the Delta band also showed significant interactions for factors Hemis versus Group [F(1, 73) = 7.2347, *p* = 0.0088] (Fig. 6e), and for factors ExpCond versus Hemis versus Group [F(2, 72) = 5.9768, *p* = 0.0040] (Fig. 6f).

In the Theta band significant interactions for factors Hemis versus Group [F(1, 73) = 5.1700, *p* = 0.0259] (Fig. 2c), and ExpCond versus Hemis versus Group [F(2, 72) = 5.0017, *p* = 0.0092] (Fig. 6d) were detected. In the control group values of coherence were significantly higher for the right hemisphere, while the ASD group did not show any significant differences (Fig. 6c). Similar results were observed for coherence values in the Delta band (Fig. 6e). Coherence values during the experimental conditions V–A and VwA were significantly lower in both groups in comparison with control condition. In the autistic group coherence during the VwA condition were significantly lower than those in the V–A experimental condition in the right hemisphere. Interhemispheric coherence values were only significantly higher in the right hemisphere in the control group, during the experimental conditions V–A and VwA (Fig. 6d).

Coherence values in the left hemisphere were significantly higher in the ASD group for all the experimental conditions. In the right hemisphere those differences were not found, but in the group of children with autism the coherence values were significantly incremented for V–A versus VwA conditions (Fig. 6f).

#### Intrahemispheric Long-Range Coherence

The Between factor Group was significant for the Delta band [F(1, 173) = 16.5465, *p* = 0.000…,] the alpha band [F(1, 173) = 4.0309, *p* = 0.0462], the Sigma band [F(1, 173) = 12.8730, *p* = 0.0004], the beta band [F(1, 173) = 125.2655, *p* = 0.000…], and the Gamma band [F(1, 173) = 33.4052, *p* = 0.000…]. For the Theta band, the factor Group did not show statistical differences [F(1, 173) = 3.1559, *p* = 0.0774].

**Fig. 7:**
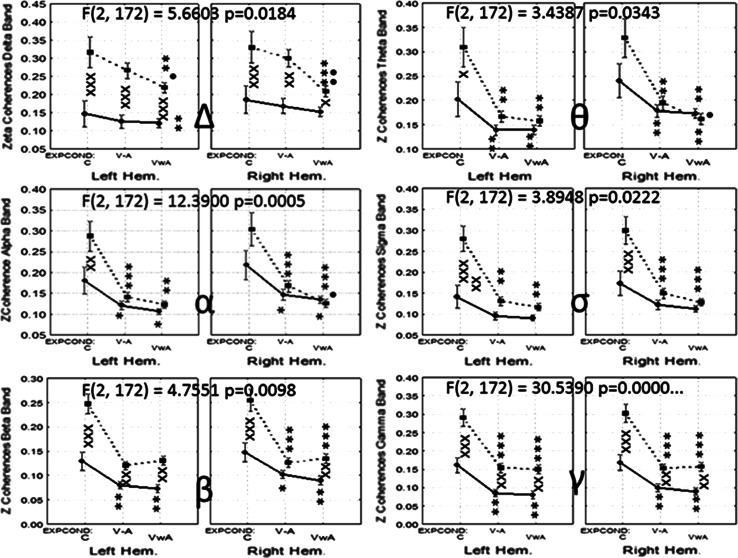
Collage of images showing the statistical analysis of the Zeta values of the long-range coherence in both groups. *Symbols* x, xx, xxx *p* < 0.05, *p* < 0.01, *p* < 0.001 for comparisons between groups; *, **, *** *p* < 0.05, *p* < 0.01, *p* < 0.001 for comparisons between experimental conditions versus control; •, •• *p* < 0.05, *p* < 0.01 for comparisons between V–AA versus VwA

Significant interactions between the Within factor Hemisphere with the Between factor Group were only found for the Delta band [F(1, 173) = 5.6603, *p* < 0.01840]. Other interactions showed statistical significance, and are represented in Fig. 7.

For the Delta band coherence values were significantly higher for ASD group in both hemispheres for all experimental conditions. Coherence values in the control group, in both hemispheres, did not show significant changes in the experimental conditions. In the autistic group the values in the V–A and VwA conditions resulted, for both hemispheres, in significantly lower values than those obtained during the control condition.

In the Theta band, in both hemispheres, and in both groups, reduced values of coherence during the conditions V–A and VwA versus control measurements were shown. In the right hemisphere, children with autism showed significantly lower values during the V–A, compared with VwA condition.

For the other EEG bands, the pattern of the changes showed a similar tendency as those previously described for the Theta band. In general, coherence values were significantly higher in the autistic group (Fig. 7).

#### Transverse Intrahemispheric Coherence

The between factor Group showed only significant values for the Delta band F(1, 123) = 7.1901, *p* < 0.000…, and for the Gamma band F(1, 123) = 7.5347, *p* < 0.0069. In both cases higher values were observed in children with autism.

Significant interactions were observed between the Hemisphere factor and the Group factor consisting in significantly higher values for the right hemisphere, but only for the control group. Among the children with autism, these differences were not found. For the Delta band the statistical significance was [F(1, 123) = 5.6857, *p* < 0.0186], for the Theta band [F(1, 123) = 5.8165, *p* < 0.0173], for the alpha band [F(1, 123) = 6.0890, *p* < 0.0150], for the Sigma band [F(1, 123) = 6.6713, *p* < 0.0110], and for the beta band [F(1, 123) = 4.6760, *p* < 0.0325]. Interactions for the factor SAreas versus Group were only detected for the Gamma band [F(1, 123) = 4.0060, *p* < 0.0475], and consisted of an increment of the coherence values found only in the control group, between the anterior versus the posterior areas.

**Fig. 8:**
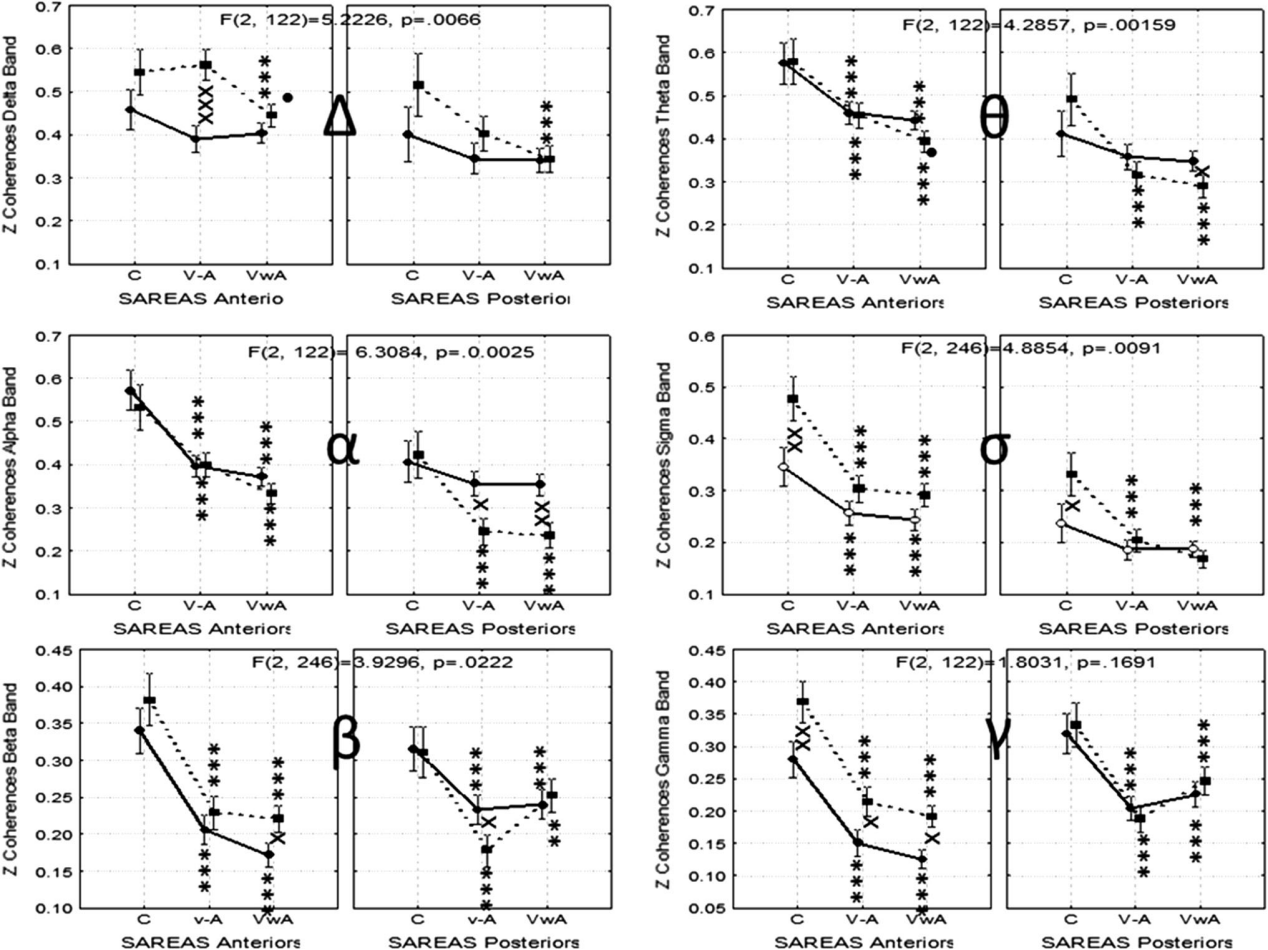
Collage of images showing the statistical analysis of the Zeta values of the intrahemispheric transverse coherence in both groups. *Symbols* x, xx, xxx *p* < 0.05, *p* < 0.01, *p* < 0.001 for comparisons between groups; *, **, *** *p* < 0.05, *p* < 0.01, *p* < 0.001 for comparisons between experimental conditions versus control; •, •• *p* < 0.05, *p* < 0.01 for comparisons between V–A versus VwA conditions

In general, coherence values showed a tendency to reduced values during the experimental conditions V–A and VwA, compared to the control condition, mainly in the anterior areas. Differences between groups were not frequent (Fig. 8).

#### Interhemispheric Coherence

The Factor Group resulted significant differences for the coherence in the EEG bands Delta [F(1, 48) = 4.8651, *p* < 0.0322], Sigma [F(1, 48) = 14.1852, *p* < 0.0004], Beta [F(1, 48) = 31.6167, *p* < 0.000…], and Gamma [F(1, 48) = 28.7894, *p* < 0.000….]. Invariably, in all cases coherence values were higher in the autistic group.

**Fig. 9:**
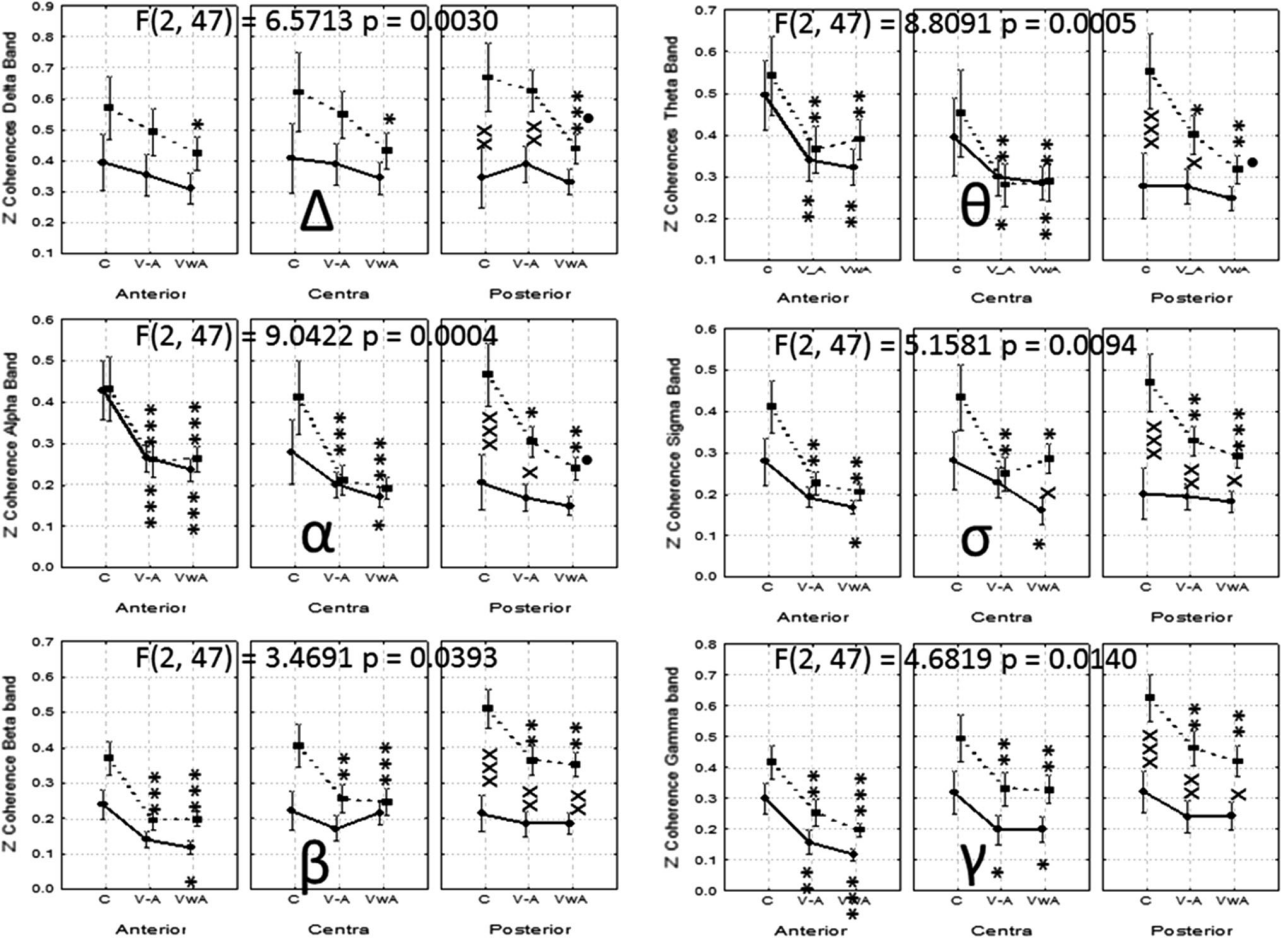
Dynamics of Zeta values for Inter-hemispheric coherence observed in both groups. *Symbols* x, xx, xxx *p* < 0.05, *p* < 0.01, *p* < 0.001 for comparisons between groups; *, **, *** *p* < 0.05, *p* < 0.01, *p* < 0.001 for comparisons between experimental conditions versus control; •, •• *p* < 0.05, *p* < 0.01 for comparisons between V–A versus VwA conditions

Significant interactions between SAreas, Hemisphere and the between factor Group are shown in the Fig. 9. In general the more definite changes were detected in the posterior EEG regions, and the coherence values in the ASD group, as a rule, were always significantly higher than those observed in the control participants in the three experimental conditions. Sometimes, these values resulted in statistically significant results and sometimes not, but that was the general tendency in all EEG bands (Fig. 9). In the Delta and beta bands, the values observed for the control group did not change during the experimental conditions, while in the ASD group dynamic changes were observed.

## Discussion

Our autistic children clearly showed a tendency to present for both absolute and normalized PSD, significant reduction in the Alpha band, and an increment in the slow Delta and the fast beta and Gamma bands, compared to the control group, in the resting, control condition. No significant differences were found for the control and the ASD groups, considering the hemisphere factor.

Alpha-band oscillations seem to be reflective of an attentional suppression mechanism when objects or features need to be specifically ignored or selected against (Banerjee et al. 2011; Fiebelkorn et al. 2012; Foxe and Snyder 2011). Several hypotheses on the generation of Alpha rhythm have been reported since several decades ago (Andersen et al. 1968; Lopes da Silva and van Leeuwen 1969; Lopes da Silva et al. 1980, 1997; de Munck et al. 2009).

Several authors have reported a reduction of Alpha activity in autism (Cantor et al. 1986; Cantor and Chabot 2009; Dawson et al. 1995). Chan reported that ASD individuals demonstrated significantly lower relative Alpha and higher relative Delta and Delta–Alpha ratio. This author considered that such abnormality in relative Alpha among ASD children is not restricted only to a single and specific location of the brain but on the contrary is a widespread pattern across the brain, possibly reflecting the neurophysiological abnormality associated with ASD (Chan et al. 2007).

We recorded EEG in an eyes-open condition which might partially explain a reduction in Alpha activity, but the absolute and normalized PSD reduction in our autistic participants was significantly different to the Control group (Barry et al. 2009). As we will discuss later, the possibility whereby all information in one’s sensory domain is processed to the level of consciousness, is lessened in ASD and might be the reason for an impaired generation of Alpha activity in autism (Markram and Markram 2010).

Exaggerated slow EEG wave activity within the Delta band is a common finding in brain injury (Nuwer et al. 2005). We found a significant increment of Delta activity in our ASD group. Although none of our children with autism showed gross anatomical abnormality in neuroimage studies, neuropathological observations in autism have reported, among other findings, features of cortical dysgenesis or migration disturbances (Casanova et al. 2006; Ozgen et al. 2011; Palmen et al. 2005, 2006; Santos et al. 2011; Schmitz et al. 2005; van Kooten et al. 2008). This might explain the significant increment of slow activity in our cases.

An interesting finding in our cases was the significant increment of activity within the fast beta and Gamma bands. Other authors have also reported an increment of high frequency EEG oscillations in children with autism (Orekhova et al. 2008; Stroganova et al. 2011).

Several authors have proposed that high frequency rhythms (12–80 Hz) are generated in neuronal networks connecting excitatory pyramidal cells and inhibitory Gamma-aminobutyric acid (GABA)-ergic interneurons (Haenschel et al. 2000; Kopell et al. 2000; Traub et al. 2000). Gamma-frequency synchronization between neural assemblies was suggested to play a role in integration of sensory information, and beta oscillations are currently considered not limited to the motor system, but more generally are involved in sensorimotor integration and top-down signaling (Wang 2010). Hence, taking in consideration the importance of the of high frequency EEG rhythms for integration of sensory information, and other perceptual and cognitive processes, this increment of fast EEG frequency oscillations in children with autism might be due to an imbalance in the excitation–inhibition regulation in the cortex and its connections with other brain structures (Orekhova et al. 2007).

Other interesting results to discuss were the findings of the EEG dynamics between experimental conditions. In general both V–A and VwA experimental conditions showed significant changes versus the control condition, for several EEG bands in both control and autistic groups. In general, spectral changes in the ASD group were more evident than in control subjects.

Nonetheless, the most interesting results came when comparing the V–A versus VwA experimental conditions. Significant reductions were found for PSD absolute values in the central region reductions for the Delta and Theta, and in the posterior region for the Sigma and beta bands, lateralized to the right hemisphere.

Nonetheless, other result very interesting comes from the EEG spectral analysis of the midline region. Again, when comparing V–A versus the VwA experimental conditions we found a significant decrement of PSD absolute values of Delta, Theta and Alpha, but an increment in the faster frequencies of the beta and Gamma bands.

Several authors have identified a number of regions, including the ventromedial prefrontal cortex (vmPFC) amygdala and ventromedial prefrontal cortex, located in the medial regions of both hemispheres, which have been identified as functional units within the of the central autonomic network, closely linked to emotion regulation (Luecken et al. 2005; Thayer et al. 2012). Dawson has indicated that both the medial temporal lobe and ventromedial prefrontal cortex have been implicated in autism. This author has emphasized that the ventromedial prefrontal cortex function is essential for generalizing and inhibiting stimulus reward associations, and therefore a dysfunction of this region might explain early joint attention impairments in autism (Dawson et al. 2001).

Hence, taking in consideration that midline leads mainly record bioelectrical activity from the medial regions of both hemispheres, when comparing V–A versus the VwA experimental conditions might induce changes in EEG spectral components in autistic kids probably related to functional changes in those areas of the central autonomic network from the medial regions of both hemispheres.

Regarding our coherence results, we recorded EEG in an eyes-open resting state and we found that children with autism showed significant higher intrahemispheric long-range coherence in the left hemisphere compared to control participants. In fact, the ASD group did not show significant differences comparing both hemispheres for the resting control condition.

On the contrary, Coben et al. (2008) recorded EEG during an eyes-closed resting condition and found underconnectivity in children with autism compared to controls, characterized by decreased intrahemispheric Delta and Theta coherence across short to medium and long inter-electrode distances. These authors also described an inter-hemispheric reduction of Delta and Theta coherence across the frontal region, and also a decrease of Delta, Theta, and Alpha coherence over the temporal regions. Murias et al. (2007a, b) assessed EEG coherence with high-density electrodes in the eyes-closed resting state and found locally elevated coherence in children with autism in the Theta frequency range, especially within left hemisphere for frontal and temporal regions. In the lower Alpha range (8–10 Hz), they found globally reduced coherence in children with autism within frontal regions and between frontal and all other scalp regions. Meanwhile Sheikhani et al. (2012). recording EEG in a calm state, awake relaxed eye-opened condition in 17 subjects with ASD, ranging in age from 6 to 11 years, reported bilaterally increased coherence in the Gamma band, especially involving the temporal lobes.

Another important result to discuss is that the ASD group subjects in general presented significant incremented coherence values for all EEG bands and for both hemispheres, compared with the control group.

Several authors have affirmed that autism arises from the development of abnormal neural networks that exhibit synchronization (Geschwind 2007; Geschwind and Levitt 2007; Gutierrez et al. 2009). This period of abnormal brain overgrowth, largely concludes before the end of the second year of life. Courchesne et al. (2011) have emphasized that by 2–3 years of age, 90 % of autistic children had brain volumes that exceeded normal average toddlers. Autistic kids present the most extreme enlargement of gray and white matter volume in frontal and temporal lobes, with an altered white matter maturation trajectory during childhood. This evidence suggests the possibility that the same regions that experience the greatest amount of early overgrowth also show the greatest aberrations in white matter with abnormally increased white matter frontal and temporal volumes and with concomitant augmentation of gray matter size in parietal, inferior temporal and superior occipital lobes. Hence, this overgrowth of white matter in autistic children might explain the significant incremented coherence values for all EEG bands and for both hemispheres, compared with the control group (Courchesne et al. 2011; Courchesne and Pierce 2005).

Duffy and Als (2012) described a stable pattern of EEG spectral coherence distinguishing children with autism from neuro-typical controls in a large case control study. These authors demonstrated reduced short-distance and reduced, as well as increased, long-distance coherences for the ASD-groups, when compared to the controls. They hypothesized that short-distance coherences may indicate poor local network function; meanwhile augmented long-distance coherences may represent compensatory processes or reduced neural lopping.

Overall, and for the four types of coherence calculations, children with autism and control participants tended to show lower coherence values for V–A and VwA compared to the control condition. Nonetheless, the most interesting finding was that when comparing the two experimental conditions, V–A versus VwA, no significant differences were found for the control group, but in the ASD group the VwA, compared with the V–A condition, had a tendency to show lower coherence values in the right hemisphere. These significant EEG hemispheric coherence differences were not found among the ASD group in the control condition, and significant differences only appeared when comparing V–A versus VwA experimental conditions.

Numerous reports have affirmed that children with autism show a tendency in responding to only one aspect of a multisensory object (Koegel 2011; Lovaas et al. 1979; Lovaas and Schreibman 1971). Therefore, several authors have affirmed that low-level auditory-visual integration is common in ASD (van der Smagt et al. 2007), and most authors report that children with autism show a dominance of somatosensory and visual over auditory stimuli (Allenet al. 2009a, b; Heaton 2009; Russo et al. 2010).

Regarding auditory stimuli, several reports suggest that in autism, a dysfunction at preconscious stages of cortical auditory discrimination occurs, playing a crucial role in the anomalous processing of auditory sensory stimuli (Foss-Feig et al. 2010; Kwakye et al. 2011; O’Connor 2012; Ouimet et al. 2012). Therefore, although atypical sensory-based behaviors are a ubiquitous feature for sensory information (Cascio et al. 2012; Magnee et al. 2008), processing of auditory sensory stimuli seems to be more impaired than visual information in autistic (Foss-Feig et al. 2010; Kwakye et al. 2011; O’Connor 2012; Ouimet et al. 2012).

Hence, we might speculate that both EEG spectral and coherence results in our children with autism when comparing V–A versus VwA experimental conditions are related to a failure of visual-auditory sensory integration, which is lateralized to the right hemisphere in the ASD group.

Nonetheless, it is important and interesting to remark that when the cartoon audio band was muted, our examiners did not detect any facial or other emotional behavioral signs among the children with autism, contrary to control participants. Further research is needed combining EEG record and autonomic assessment by heart rate variability, for comparing the effects of our experimental conditions in children with autism.

One limitation of this paper is the relative small number participants, although a detailed statistical power analysis processing demonstrated that these results were reliable.

It is necessary to remark again that for comparing our results with other publications, it is necessary to take in consideration that we recorded EEG in an eye-open state, and we used three different experimental conditions. Nonetheless, regarding QEEG spectral analysis, our results allowed to clearly differentiate children with autism from control subjects, and are in general in concordance with the reports of other authors (Cantor et al. 1986; Cantor and Chabot 2009; Chan and Leung 2006; Chan et al. 2007; Coben et al. 2008; Ogawa et al. 1982). The augmented coherence values in children with autism compared to control subjects probably reflect the existence of rigid neuronal networks which might explain the classical manifestation of repetitive behavior expressions, and impairments in social interaction, communication and imagination, which characterized autism (Belmonte et al. 2009; Gadow and Drabick 2012; Rapin and Tuchman 2008; Silver and Rapin 2012; Zappella 2012).

Our experimental design allowed us to find EEG changes, both in spectral and coherence analysis, which might be related to a failure of visual-auditory sensory integration, which is lateralized to the right hemisphere in children with autism. Therefore, the usage of the three experimental conditions or our methodological design demonstrated to be an useful paradigm to differentiate autistic children from normal subjects, and should be used in future research in autism.
